# Multi-Omics Revealed Regulatory Mechanisms Underlying the Flowering of *Ferula sinkiangensis* across Three Dimensions

**DOI:** 10.3390/genes15101275

**Published:** 2024-09-28

**Authors:** Congzhao Fan, Yanfei Li, Jizhao Zhang, Yaqin Zhao, Yigong Zhang, Jun Zhu, Xingwang Gao, Yan Liang, Yuanjin Qiu, Jingyuan Song, Guoping Wang

**Affiliations:** 1 Key Laboratory of Chinese Medicine Resources Conservation, State Administration of Traditional Chinese Medicine of the People’s Republic of China, Institute of Medicinal Plant Development, Chinese Academy of Medical Science & Peking Union Medical College, Beijing 100193, China; fcz_840701@126.com (C.F.); muziyanfei@126.com (Y.L.); songjingyuan@hotmail.com (J.S.); 2 Xinjiang Key Laboratory of Chinese Materia Medica and Ethnic Materia Medica, Xinjiang Institute of Chinese Materia Medica and Ethnical Materia, Urumqi 830011, China; jizhao0214@163.com (J.Z.); xjzyq123@126.com (Y.Z.); zhujun1hao@163.com (J.Z.); cxmqyj@189.cn (Y.Q.); 3 Xinjiang Key Laboratory of Biological Resources and Genetic Engineering, College of Life Science, and Technology, Xinjiang University, Urumqi 830017, China; zhangyg@xju.edu.cn (Y.Z.); gxw@xju.edu.cn (X.G.); 107552301023@stu.xju.edu.cn (Y.L.)

**Keywords:** *Ferula sinkiangensis*, flowering, transcriptome, proteome, metabolome

## Abstract

**Backgroud/Objectives:** Ferula spp. is an essential crop in Central Asia with pronounced economic benefits governed by its flowering process. However, the mechanisms of the flowering phenotype remain unclear. **Methods:** In this study, using *F. sinkiangensis* as a model plant, we integrated transcriptome, proteome, and metabolome analyses to compare the multilayer differences in leaves and roots of plants with flowering and unflowering phenotypes. **Results:** We found that several variations in the transcriptome, proteome, and metabolome were closely associated with flowering. The Photosynthesis and Phenylpropanoid biosynthesis pathways in plants with the flowering phenotype were more active. Additionally, three flowering genes, named *FL2–FL4*, were upregulated in the leaves of flowering plants. Notably, six transcription factors were potentially responsible for regulating the expression of *FL2–FL4* in the leaves to mediate flowering process of *F. sinkiangensis*. Moreover, genes relevant to Photosynthesis and Phenylpropanoid biosynthesis were also involved in regulating the expression of *FL2–FL4* in flowering plants. **Conclusions:** The active regulation network together with Photosynthesis and Phenylpropanoid biosynthesis were essential for inducing the expression of flowering-related genes in leaves to promote the flowering process of *F. sinkiangensis*.

## 1. Introduction

*F.* spp. is a member of the Apiaceae family and widely grown in the Mediterranean, Central Asia, and the Middle East [1]. As herbaceous perennial plants, the *F*. species are critical economic crops in Central Asia as their roots can produce resins, which are used as important natural medicine in many countries [2,3]. Typically, *F. sinkiangensis* and *F. fukanensis* were used as traditional herbs in Xinjiang, China [3]. With increasing demands for natural products, *F. sinkiangensis* has attracted great attention in terms of economic value due to its specific metabolic composition. Asafoetida gum is the primary product of *F. sinkiangensis*, which determined its economic value and medicinal quality [3]. It was found that Asafoetida gum from *F. sinkiangensis* exerted numerous medicinal bioactivities for improving nervous disorders, respiratory problems, gastrointestinal disorders, dramatic inflammation, and metabolic disorders in clinic [4]. Typically, Asafoetida gum is produced during the flowering process [1], thus the flowering trait of *F. sinkiangensis* directly influenced the market supply and economic quality of *F. sinkiangensis*. It is necessary to explore the molecular mechanism of flowering of *F. sinkiangensis* to ensure its economic production and medicinal quality.

The regulation of the flowering phenotype is complex and is affected by numerous factors in plants [5]. For instance, amino acid metabolism in the roots is essential for plant development, especially when regulating flowering [6]. Typically, amino acid metabolism in the root system is an important component of nitrogen metabolism, signaling, and Hormone synthesis [6,7]. These procedures can modulate flowering timing and development by influencing nutritional features, hormone balance, and gene expression [6,7]. Nitrogen metabolism is closely tied to plant growth and development, especially in determining flowering time and resource dispersal [6]. Moreover, amino acid metabolism is associated with plant Hormone synthesis, including auxin, cytokinins, and gibberellin, which are important in regulating plant flowering [8]. For instance, as precursors of auxin and cytokinins, tryptophan and glutamine indirectly affect plant flowering timing by regulating gene expression and cell division [9,10]. Additionally, certain amino acids can operate as signaling molecules, activating or inhibiting transcription factors that regulate the expression of flowering genes [6,7]. Root amino acid metabolism directly affects the expression of flowering-related genes, including *FT* genes [6,11]. In addition to amino acids, plant Photosynthesis regulates plant flowering by providing energy and carbon sources for plant development [12]. The improvement of photosynthetic efficiency is closely tied to the early flowering of plants [12]. In *Arabidopsis thaliana*, sucrose accumulation during Photosynthesis induced the expression of the flowering gene *FT*, promoting flowering [13]. Additionally, Phenylpropanoids regulate plant development, including flowering traits [14]. Recently, flavonoid roles in regulating flowering were characterized in numerous plants. Phenylpropanoids can influence flowering by regulating the transport of growth hormones (including auxin) [15]. In *Petunia*, mutations in the Flavonoid biosynthesis pathway caused abnormal auxin distribution, delaying flowering time [15]. Furthermore, in *A. thaliana*, overexpression of the *phenylalanine ammoniase* (*PAL*) gene caused changes in endogenous hormone levels, including gibberellin and abscisic acid, affecting the flowering process [16]. However, the mechanisms of flowering in *F. sinkiangensis* are still not clear.

In this study, we studied the flowering trait of *F. sinkiangensis*. Multi-omics analysis, including transcriptome, proteome, and metabolome, was deployed on leaves and roots of flowering and unflowering plants to examine the mechanisms underlying the flowering phenotype of *F. sinkiangensis*. We found that Photosynthesis and Phenylpropanoid biosynthesis were associated with the flowering phenotype in *F. sinkiangensis*. Six transcription factors were identified to regulate the expression of flowering genes in leaves, which positively impact the flowering process of *F. sinkiangensis*. Thus, our work investigated the molecular mechanism of flowering, providing a theoretical basis for dealing with the difficulty in flowering, which can promote the protection and sustainable utilization of *F. sinkiangensis* resources.

## 2. Materials and Methods

### 2.1. Sample Collection and Preparation

*F. sinkiangensis* samples were obtained from the Nurturing Base of the Xinjiang Institute of Chinese Materia Medica and Ethnical Materia in Yining County (82.0574° E, 43.6743° N), Xinjiang, China. Leaves and roots from three flowering and non-flowering *F. sinkiangensis* plants were collected, flash-frozen in liquid nitrogen, and stored at −80 °C. These samples included FLL (flowering leaves), FLR (flowering roots), UFLL (non-flowering leaves), and UFLR (non-flowering roots). This work complied with all required institutional, national, and international guidelines. Specimens were identified by Wang Guoping and were deposited in the Xinjiang Institute of Chinese Materia Medica and Ethnical Materia (Urumqi, Congzhao Fan, fcz_840701@126.com) under the voucher number 654021120525001LY.

### 2.2. RNA Extraction and Transcriptome Sequencing

Total RNA from three replicates of *F. sinkiangensis* in individual experimental clusters was isolated with TRIzol (No.10296010CN, USA) based on the manufacturer’s directions [17]. After characterizing the RNA quality and quantity, all samples were pooled to conduct Illumina RNA sequencing at Biotree. In total, 1.5 µg RNA in each isolate was employed in library preparation after isolating polyA RNA, and the cDNA libraries were produced utilizing a NEBNext^®^ Ultra™ RNA Library Prep Kit for Illumina^®^ (No.E7530L, USA) adhering to the company’s directions. The AMPure XP system (Beckman Coulter, Beverly, MA, USA) was employed to isolate 250~300 bp cDNA for subsequent sequencing, and an Agilent Bioanalyzer 2100 (No.Agilent 2100, Santa Clara, CA, USA) was used to assess the quality of the library. The samples were subject to RNA-Seq using the Illumina Hiseq4000 platform (San Diego, CA, USA). Raw data (raw reads) in fastq format were processed to produce high-quality clean data (clean reads). Further transcriptome assembly was conducted with Trinity.

### 2.3. Metabolite Extraction

Three biological replicates of *F. sinkiangensis* in each cluster were harvested for metabolite isolation for metabolic composition analysis in flowering and unflowering *F. sinkiangensis*. In total, 20 mg from each isolate from FLL, FLR, UFLL, and UFLR samples were employed and underwent extraction using 1000 μL of extract solution (methanol:water = 3:1, with an isotopically labeled internal standard) through ultrasonic extraction three times. Following centrifugation, the supernatant from the quality control (QC) sample and each experimental extract were harvested for subsequent LC-MS/MS assessment [18].

### 2.4. LC–MS/MS Analysis and Data Processing

A UHPLC system (Vanquish, Thermo Fisher Scientific, Waltham, MA, USA) utilizing a UPLC HSS T3 column (2.1 mm × 100 mm, 1.8 μm) linked to an Orbitrap Exploris 120 mass spectrometer (Orbitrap MS, Thermo) was employed for LC-MS/MS analysis. Solvents consisted of 5 mM ammonium acetate and 5 mM acetic acid mixed with water (A) and acetonitrile (B) as mobile phases. The injection volume and temperature were established at 2 μL and 4 °C, respectively. The information-dependent acquisition (IDA) mode was employed to obtain MS/MS spectra and assess the complete MS spectrum. The ESI source conditions were established as follows: The capillary temperature was 320 °C, the full MS resolution was 60,000, the MS/MS resolution was 15,000, the collision energy was 10/30/60 in NCE mode, and the spray Voltage was 3.8 kV (positive) or −3.4 kV (negative), respectively.

Raw data from all samples were processed using an in-house MS2 database (BiotreeDB) for metabolite characterization [19]. The cutoff for annotation was established at 0.3. Typically, *p* < 0.05 and fold change > 2.0 were employed to characterize differential metabolites. Further metabolomics analysis was conducted using Metaboanalyst 3.0, encompassing PCA, OPLS-DA, and PLS-DA algorithms, as well as KEGG pathway enrichment evaluation [20].

### 2.5. Proteome Sequencing Analysis

All *F. sinkiangensis* samples with three biological replicates were harvested for protein extraction. Protein extraction and sample analysis were undertaken with SDT (4% SDS, 1 mM DTT, 100 mM Tris-HCl, pH 7.6) buffer and quantified using a BCA Protein Assay Kit (BioRad, Hercules, CA, USA). The protein was digested utilizing trypsin and desalted with C18 cartridges (Empore™ SPE Cartridges C18 (standard density), bed I.D. 7 mm, volume 3 mL, Sigma, Burlington, MA, USA), concentrated through vacuum centrifugation and resuspended with 40 µL of 0.1% (*v*/*v*) formic acid. After quality assessment, the protein samples were labeled and separated via high pH reverse-phase liquid chromatography, followed by detection using an OrbitrapFusion mass spectrometer(Orbitrap Fusion, FIS, USA). Mass spectrometry results were obtained using Maxquant (v1.5.2.8), and retrieval characteristics were established; the database included the *F. sinkiangensis* proteome sequences. The minimum length of the peptide was established at seven amino acid residues. The maximum number of peptide modifications was set as five. The mass error tolerance of the first search and main search was set to 20 ppm and 5 ppm for the primary parent ion, respectively, and 0.02 Da for the secondary fragment ion. The FDR for protein identification and PSM identification was established at 1%. Log1.2foldchange and *p* < 0.05 were employed as thresholds to identify differentially expressed proteins (DEPs) [21,22,23].

### 2.6. Proteome-Associated LC-MS/MS Detection and Analysis

For each sample, 200 ng of total peptides were separated and examined using a nano UPLC (Evosep one) coupled to a timsTOF Pro2 instrument (Bruker) employing a nano electrospray ion source. Separation was undertaken with a reversed phase column (PePSep C18, 1.9 μm, 150 μm × 15 cm, Bruker, Karlsruhe, Germany). Mobile phases consisted of H_2_O with 0.1% FA (phase A) and CAN supplemented with 0.1% FA (phase B). Separation of samples was executed using a 44-min gradient. The mass spectrometer utilized DDA PaSEF (Brooke Company, Steißlingen, Germany) mode for DDA data acquisition, and the scanning range ranged from 100 to 1700 m/z for MS1. During PASEF MS/MS scanning, the impact energy increased linearly with ion mobility, from 20 eV (1/K0 = 0.6 Vs/cm^2^) to 59 eV (1/K0 = 1.6 Vs/cm^2^).

### 2.7. Bioinformatic Analysis

Fragments Per Kilobase of exon model per Million mapped fragments (FPKM) was utilized to assess gene expression levels via the DESeq2 R package (1.10.1) [24]. Genes possessing an adjusted *p*-value < 0.05 and Fold change > 2.0 were rec differentially expressed genes (DEGs). Gene ontology (GO) terms were mapped, and sequences were annotated with Blast2GO 6.0. The GO annotation findings were presented using R package GOplot 1.0.2 [25]. BLAST2GO 6.0 software was employed for functional annotation following Ye et al. [26]. The DEPs and DEGs in each comparison were mapped according to the Kyoto Encyclopedia of Genes and Genomes (KEGG) database (http://www.genome.jp/kegg, accessed on 31 July 2022) [27]. GO and KEGG pathway analyses were undertaken with clusterprofiler 2.0. Only functional categories and pathways with *p*-values below 0.05 were significant. WGCNA analysis was conducted following the procedure of Du et al. [28].

## 3. Results

### 3.1. Transcriptome Variations in Leaves and Roots Associated with the Flowering Phenotype

**Figure 1:**
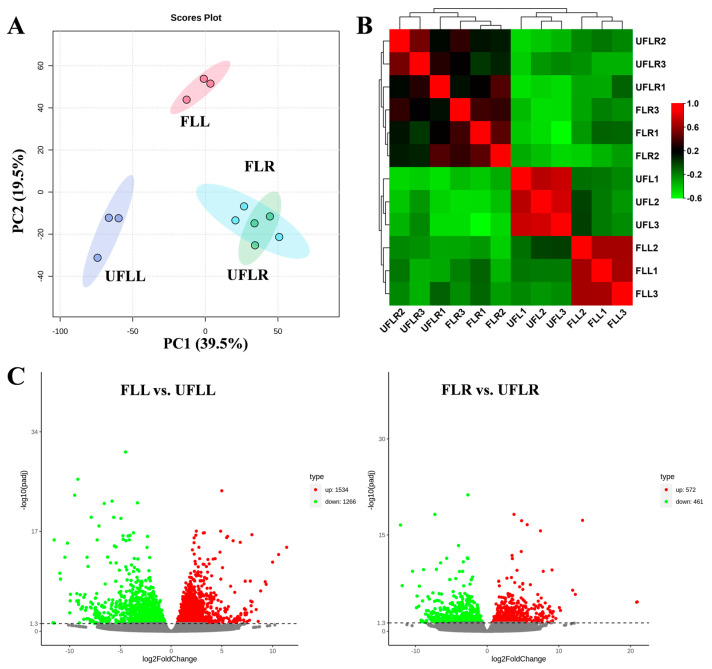
Transcriptional landscape in leaves and roots of flowering and unflowering *F. sinkiangensis*. (**A**): PCA score plots of samples depicting pronounced differentiation across FLL and UFLL at the transcriptional level. The ellipse indicates the 95% confidence interval. (**B**): Unsupervised correlation assessment of all transcriptome profiles in flowering and unflowering *F. sinkiangensis* according to the Pearson algorithm. High and low correlation relationships were presented in red and green, respectively. (**C**): Volcano plot indicating the differential genes (DEGs) with Log2(Foldchanges) ≥ 1.0 and *p* ≤ 0.05 in FLL vs. UFLL and FLR vs. UFLR pairwise comparisons. The upregulated genes are presented in red, and the gray points represented genes with no significance in each pairwise comparison, while downregulated representatives are illustrated in green.

To assess the underlying flowering mechanisms of *F. sinkiangensis*, we obtained the leaves and roots of flowering and unflowering plants (FLL, UFLL, FLR, and UFLR) for transcriptome analysis, using biological triplicates in each class. In total, 44,104 genes were identified in our transcriptome profiles. We conducted PCA on the transcriptome results to evaluate the transcriptional changes in leaves and roots of flowering and unflowering individuals (Figure 1A). A PCA plot was produced with PC1 and PC2, accounting for 39.5% and 19.5% of variations throughout the samples (Figure 1A). This plotted PCA examination indicated that all biological replicates of leaves from flowering and unflowering representatives (FLL and UFLL) were distributed in a single region according to their transcription pattern and separated from one another (Figure 1A), indicating that transcriptome variations in leaves encompassed in the flowering phenotype of *F. sinkiangensis*. However, the biological replicates of roots from flowering and unflowering plants (FLR and UFLR) overlapped with one another (Figure 1A), indicating that the transcriptional changes in roots were not significantly relevant to the flowering phenotype in *F. sinkiangensis*. Typically, the differences relevant to flowering phenotype were primarily identified in PC1, while PC2 predominantly explained the differences among tissues (Figure 1A). Further unsupervised relationship assessment of all transcriptome results achieved agreement with PCA results and showed that the transcriptome changes in leaves were highly correlated with the flowering phenotypes of *F. sinkiangensis*, whereas few differences were identified in the roots between flowering and unflowering *F. sinkiangensis* (Figure 1B).

To investigate the detailed changes in the transcriptome of flowering and unflowering *F. sinkiangensis*, we examined the differentially expressed genes (DEGs) in FLL vs. UFLL and FLR vs. UFLR according to *p* < 0.05 and Fold change > 2.0 (Figure 1C). In total, 2800 DEGs were found in FLL vs. UFLL, with 1534 upregulated and 1266 downregulated DEGs (Figure 1C). In addition, 1033 DEGs were identified in the FLR vs. UFLR comparison, with 572 upregulated and 461 downregulated DEGs (Figure 1C). Notably, the relatively fewer DEGs in FLR vs. UFLR compared with DEGs (2800) FLL vs. UFLL supported that the flowering mechanisms of *F. sinkiangensis* are primarily relevant to the transcription alterations in leaves but not roots. Thus, transcriptional changes are involved in the flowering mechanisms of *F. sinkiangensis*, particularly in leaves.

### 3.2. Transcriptional Changes in Phenylpropanoid, Hormones, and Photosynthesis Associated with the Flowering Phenotype

To examine the underlying flowering mechanisms of *F. sinkiangensis*, we conducted functional analysis on DEGs from FLL vs. UFLL and FLR vs. UFLR pairwise comparisons. The GO enrichment analysis on DEGs from FLR vs. UFLR indicated that these DEGs were included in 176 GO terms, with 26 GO terms significantly enriched compared to DEGs from FLR vs. UFLR (Table S1). Specifically, these DEGs from FLR vs. UFLR are mainly involved in carbohydrate metabolic process, cell wall modification, defense response, phosphatidylinositol metabolic process, response to oxidative stress, response to wounding, negative regulation of translation, methylation, carboxylic acid metabolic process, and trehalose biosynthetic process, with bioactivities of enzyme inhibitor activity, hydrolase activity, pectinesterase activity, carbohydrate binding, protein dimerization activity, catalytic activity, and transmembrane transporter activity (Table S1). Subsequent KEGG enrichment analysis of these DEGs from FLR vs. UFLR demonstrated a significant overrepresentation of 10 pathways, particularly the MAPK signaling pathway, Pentose and glucuronate interconversions, Plant hormone signal transduction, Carotenoid biosynthesis, Starch and sucrose metabolism, and Glucosinolate biosynthesis (Table S2), indicating that signaling transduction and primary metabolism in roots might also involve flowering mechanisms of *F. sinkiangensis*.

**Figure 2:**
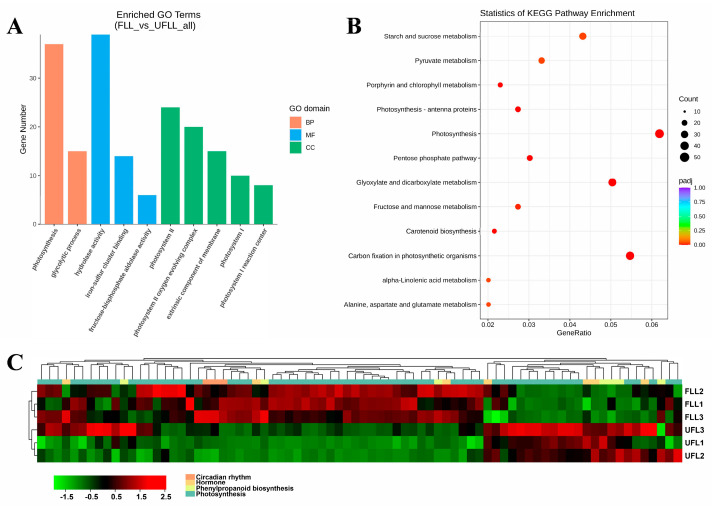
Transcription alterations of Photosynthesis and Phenylpropanoid biosynthesis in leaves involved in the flowering process of *F. sinkiangensis*. (**A**): Gene ontology (GO) enrichment analysis of DEGs from FLL vs. UFLL pairwise comparison. The terms cellular component, molecular function, and biological progress are shown in green, blue, and orange, respectively. The columns represented the number of DEGs in each GO term. (**B**): Scatter plot of the most enriched KEGG pathways of all DEGs from FLL vs. UFLL pairwise comparisons. The size and color of each plot indicate the number of genes and the significance of each associated pathway. The *x*-axis represents the richness factor of each pathway. The impact factor was produced by adding the importance measures of matched genes with all proteins in the pathway. (**C**): Heatmaps of the relative expression abundances of DEGs associated with Photosynthesis, Phenylpropanoid, and Hormone synthesis in leaves of flowering and unflowering *F. sinkiangensis*. The scale bar illustrated the average FPKM levels of DEGs from each group. The high and low expression levels were presented in red and green, respectively.

Subsequently, we examined the function of DEGs from FLL vs. UFLL and found that these DEGs mainly operated in photosystem II, photosystem II oxygen-evolving complex, the extrinsic component of membrane, photosystem I, photosystem I reaction center, and chloroplast (Figure 2A; Table S3), with bioactivities related to iron-sulfur cluster binding, hydrolase activity, fructose-bisphosphate aldolase activity, catalytic activity, metal ion binding, oxidoreductase activity, and coenzyme binding (Figure 2A; Table S2). The results relevant to the biological process indicated that these DEGs from FLL vs. UFLL are primarily involved in Photosynthesis, glycolytic process, cellular protein modification process, trehalose biosynthetic process, and metal ion transport (Figure 2A; Table S2). The Photosynthesis level was closely tied to plant flowering phenotypes, offering energy to maintain the plant flowering process. Further KEGG pathway investigation indicated that transcription changes related to Photosynthesis, Carbon fixation in photosynthetic organisms, Glyoxylate and dicarboxylate metabolism, Phenylpropanoid biosynthesis, Pentose phosphate pathway, Porphyrin and chlorophyll metabolism, Carotenoid biosynthesis, Fructose and mannose metabolism, α-Linolenic acid metabolism, Pyruvate metabolism, and Alanine, aspartate, and glutamate metabolism were the main features of FLL vs. UFLL (Figure 2B), suggesting their roles in the flowering process of *F. sinkiangensis*. Commonly, secondary metabolisms represented by Phenylpropanoid biosynthesis have also been involved in flowering mechanisms of plants. We analyzed the expression level of genes associated with these three terms in leaves of flowering and unflowering *F. sinkiangensis*. As presented in Figure 2C, most of the genes relevant to Photosynthesis and Phenylpropanoid were highly expressed in the leaves of flowering *F. sinkiangensis* compared to the leaves of unflowering *F. sinkiangensis* (Figure 2C). These results suggested that transcription upregulation is associated with primary metabolisms in roots, together with Photosynthesis and Phenylpropanoid biosynthesis in leaves, which is involved in the flowering process of *F. sinkiangensis*.

### 3.3. Proteome Profiling in Leaf and Root Associated with Flowering Phenotype

**Figure 3:**
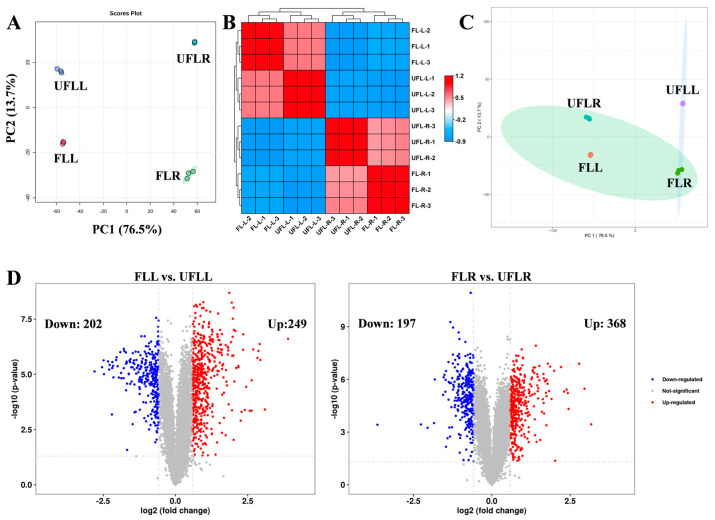
Proteome landscape in leaves and roots of flowering and unflowering *F. sinkiangensis*. (**A**): Principal component analysis of proteome data in leaves and roots of flowering and unflowering *F. sinkiangensis*. The principal component score map was developed using PC1 and PC2. The ellipse represented the 95% confidence intervals, the degree of separation of samples from each group, and the stability among biological replicates. (**B**): Correlation analysis of proteome profiles of leaves and roots of flowering and unflowering *F. sinkiangensis*. Correlations among samples were determined using the Pearson correlation coefficient (high: red; low: blue). (**C**): Self-organizing neural network analysis proteome profiles of leaf and root of flowering and unflowering *F. sinkiangensis*. The ellipse in different color represented the 90% confidence intervals. (**D**): The volcano plot presented the differentially expressed proteins in FLL vs. UFLL and FLR vs. UFLR pairwise comparisons based on Log2(Foldchange) > 1.0 and *p* < 0.05. The up- and downregulated proteins in each comparison were depicted in red and blue, respectively.

To examine the regulation mechanism of the flowering process, we collected the root and leaf tissues of flowering and non-flowering plants (FLL, UFLL, UFLR, and FLR) for proteomic sequencing. In total, 7755 proteins were identified across 12 samples. PCA plots of all proteome profiles were constructed using PC1 and PC2, which accounted for 76.5% and 13.7% of the differences in protein patterns in all samples, respectively (Figure 3A). As illustrated in Figure 1A, FLR, UFLR, FLL, and UFLL samples were significantly separated from one another according to their proteome pattern, suggesting that proteome changes in leaf and root tissues were closely associated with flowering traits of *F. sinkiangensis* (Figure 3A). Among them, PC1 primarily explained the proteome differences between flowering and non-flowering samples, whereas PC2 mainly accounted for proteome differences among different tissues, suggesting that proteome variations in leaves and roots of *F. sinkiangensis* were closely related to flowering traits, without tissue specificity (Figure 3A). Correlation analysis based on the Pearson algorithm showed that samples from FLR, UFLR, FLL, and UFLL groups were clustered on four branches, among which samples of the same tissue were clustered on the same primary branch (Figure 3B), supporting the proteome alterations in leaves and roots involved in the flowering of *F. sinkiangensis*. The correlation between biological replicates was > 80% (Figure 3B), indicating that the sequencing results were stable and could be employed for subsequent analysis to elucidate the flowering mechanism. Using a self-organizing neural network algorithm (SOM), we found that UFLL samples were spread in a single region, while FLR, UFLR, and FLL samples were clustered in another region, suggesting that flowering traits of *F. sinkiangensis* were mainly associated with proteome changes in leaf tissue (Figure 3C), aligning with transcriptome results.

Subsequently, |Log2(Foldchange)| > 1.0 and *p* < 0.05 were employed as criteria for screening differentially expressed (DEPs). Of the 565 DEPs identified in FLR vs. UFLR, 368 were upregulated and 197 were downregulated (Figure 3D). In parallel, among the 451 DEPs from FLL vs. UFLL, 249 were upregulated and 202 were downregulated (Figure 3D). Overall, the flowering traits of *F. sinkiangensis* were primarily related to the proteome changes in leaves of *F. sinkiangensis*.

### 3.4. Proteomic Changes in Photosynthesis and Phenylpropanoid Biosynthesis in Leaves Associated with the Flowering Process of F. sinkiangensis

To characterize the biological functions of flowering-related DEPs in the roots of *F. sinkiangensis*, GO enrichment analysis was conducted on 565 DEPs from FLR vs. UFLR. The results showed that 258 GO categories included DEPs from FLR vs. UFLR comparison (*p* < 0.05, Table S4). These DEPs primarily functioned in extracellular regions, cell walls, nucleosomes, chromosomes, and plastids and exerted catalytic activity, oxidoreductase activity (Table S4), hydrolase activity, cofactor binding, and galactosidase activity. The results regarding biological processes showed that these DEPs from FLR vs. UFLR are mainly involved in the carbohydrate metabolic process, cellular metabolic process, starch biosynthetic process, glucan biosynthetic process, energy reserve metabolic process, branched-chain amino acid biosynthetic process, and lipid catabolic process (Table S4). Further KEGG pathway enrichment analysis demonstrated that proteome changes involved in Amino sugar and nucleotide sugar metabolism, Phenylpropanoid biosynthesis, Starch and sucrose metabolism, and amino acid biosynthesis were the main features in FLR vs. UFLR comparison (Table S5), indicating the changes of these pathways at the protein level in roots involved in the flowering process of *F. sinkiangensis*.

**Figure 4:**
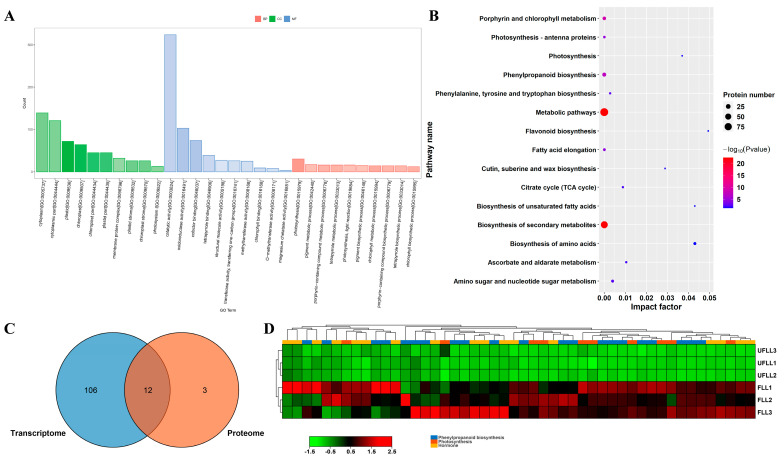
Proteome alterations in Photosynthesis and Phenylpropanoid biosynthesis in leaves involved in the flowering process of *F. sinkiangensis*. (**A**): Gene ontology (GO) enrichment analysis on DEPs from FLL vs. UFLL pairwise comparison. The terms cellular component, molecular function, and biological progress are shown in green, blue, and orange, respectively. The column represented the number of DEPs in each GO term. (**B**): Scatter plot of the most enriched KEGG pathways of all DEPs from FLL vs. UFLL pairwise comparison. The size and color of each plot denote the number of proteins and the significance of each associated pathway. The *x*−axis represents the rich factor of each pathway. The impact factor was generated by adding the importance measures of matched proteins of all proteins in the pathway. (**C**): Venn diagram of transcriptome and proteome. (**D**): Heatmaps of the relative expression abundances of DEPs relevant to Photosynthesis, Phenylpropanoid, and Hormone synthesis in the leaves of flowering and unflowering *F. sinkiangensis*. The scale bar indicated the average level of DEPs across each group. The high and low levels were presented in red and green, respectively.

We analyzed the role of 451 DEPs from a comparison between FLL vs. UFLL and found that these DEPs were significantly involved in 397 GO terms (*p* < 0.05; Figure 4A). These DEPs are primarily involved in plastid, chloroplast, plastid matrix, cytoplasm, photosystem I, and membrane protein complex (Figure 4A), with bioactivities of cofactor binding, oxidoreductase activity, catalytic activity, methyltransferase activity, O-methyltransferase activity, coenzyme binding, 6-phosphofructokinase activity, monooxygenase activity, and phosphofructokinase activity (Figure 4A). For biological processes, these flowering-associated DEPs from leaves are mainly involved in photosynthesis, chlorophyll metabolic process, porphyrin-containing compound metabolic process, tetrapyrrole metabolic process, pigment metabolic process, cofactor metabolic process, organic acid biosynthetic process, small molecule biosynthetic process, protein-chromophore linkage, and monocarboxylic acid biosynthetic process (Figure 4A). KEGG analysis of these DEPs showed significant enrichment of Porphyrin and chlorophyll metabolism, Phenylpropanoid biosynthesis, photosynthesis, Fatty acid elongation, Phenylalanine, tyrosine and tryptophan biosynthesis, Amino sugar and nucleotide sugar metabolism, Citrate cycle (TCA cycle), Ascorbate and aldarate metabolism, Hormone signal transduction, Biosynthesis of unsaturated fatty acids, Biosynthesis of amino acids, and Flavonoid biosynthesis (Figure 4B). Consistent with transcriptome results of leaves, Photosynthesis and Phenylpropanoid biosynthesis were the major metabolic drivers of change at the proteome levels in the leaf, relevant to the flowering traits of *F. sinkiangensis* (Figure 4B,C), suggesting their importance in the flowering traits of *F. sinkiangensis*. Notably, most proteins involved in Photosynthesis and Phenylpropanoid biosynthesis were also highly expressed in the leaves of flowering plants, compared to unflowering plants (Figure 4D), indicating that the activation of these processes contributed to the flowering process of *F. sinkiangensis*.

### 3.5. Metabolome Profiling Unveils That Phenylpropanoid Accumulation Is Associated with the Flowering Process

**Figure 5:**
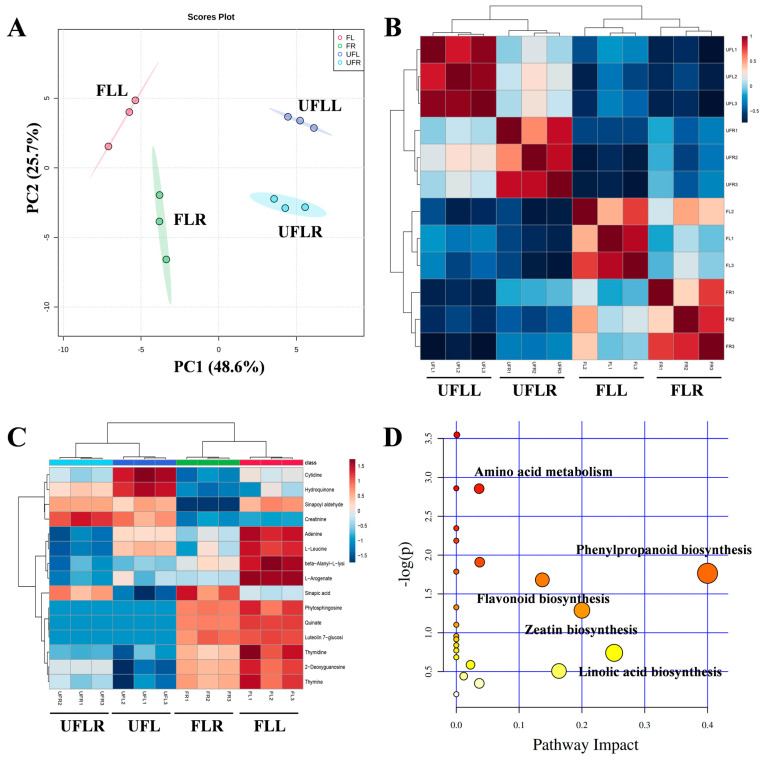
Metabolic landscape in roots and leaves of flowering and unflowering *F. sinkiangensis*. (**A**): Analysis using PCA scores plot of the samples indicates a distinct separation between FLL, FLR, UFLR, and UFLL at the metabolome level, with the ellipse representing the 95% confidence interval. (**B**): Unsupervised hierarchical clustering analysis reveals the differentiation in metabolic patterns among the FLL, FLR, UFLR, and UFLL groups. (**C**): A heatmap demonstrates the varying levels of Phenylpropanoids and flavonoids in these groups. The up- and downregulated metabolites were presented with red and blue, respectively. The normalized peak area of metabolites from each sample was utilized to denote the abundances. (**D**): KEGG pathway enrichment analysis on DEMs among FLL, FLR, UFLR, and UFLL groups. Different color levels indicated varying levels of significance in metabolic pathways, ranging from low (green) to high (red). The scatter size denoted the metabolite number from each pathway.

To examine the metabolic alterations linked to the flowering of *F. sinkiangensis*, all samples used for transcriptome sequencing were collected for metabolic analysis. In total, 863 metabolites were identified in the leaves and roots of *F. sinkiangensis*. Further PCA analysis on all metabolic profiles indicated that FLL, FLR, UFLR, and UFLL groups were separated from one another, demonstrating that metabolic changes were involved in the flowering trait of *F. sinkiangensis* (Figure 5A). Similarly, unsupervised correlation analysis on metabolic profiles showed that the metabolic makeup significantly differed among FLL, FLR, UFLR, and UFLL groups (Figure 5B), supporting the notion that the metabolic changes were involved in the flowering of *F. sinkiangensis*. The differentially expressed metabolites (DEMs) among all samples were filtered using VIP > 1.0. KEGG enrichment assessment of selected DEMs demonstrated that Phenylpropanoid biosynthesis, Purine metabolism, Sphingolipid metabolism, Zeatin biosynthesis, linolic acid biosynthesis, and Pyrimidine metabolism were significantly enriched (Figure 5D). Typically, Phenylpropanoid biosynthesis was the most significant pathway across all groups, demonstrating its importance in the flowering of *F. sinkiangensis* (Figure 5D). We identified that the Phenylpropanoid and flavonoid levels were accumulated in FLL samples compared to other groups (Figure 5C), suggesting the accumulation of Phenylpropanoid might contribute to the flowering of *F. sinkiangensis*. Numerous studies have shown that the increases in Phenylpropanoids positively affect the flowering of plants. Overall, these findings demonstrated that the accumulation of Phenylpropanoids and flavonoids in leaves was necessary for the flowering of *F. sinkiangensis.*

### 3.6. Three Genes Promote Flowering in F. sinkiangensis

**Figure 6:**
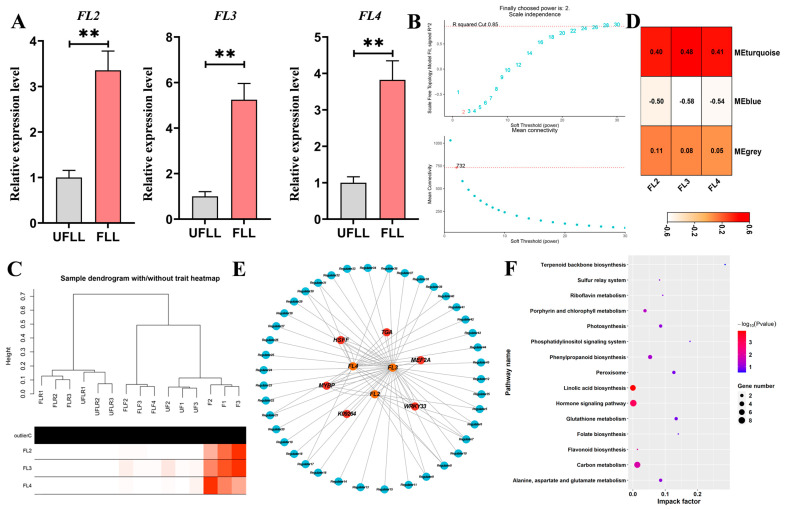
Transcription factors, Phenylpropanoid biosynthesis, Photosynthesis, and Linolic acid biosynthesis constructed the regulatory network of flowering-related genes in *F. sinkiangensis*. (**A**): RT-qPCR detection of the expression levels of genes relevant to flowering traits in *F. sinkiangensis*. “**: *p* < 0.01 according to Student’s *t*−test”. (**B**): Scale independence and mean connectivity analysis of WGCNA construction. (**C**): Sample clustering according to the expression patterns of *FL2–FL4* in *F. sinkiangensis*. (**D**): Heatmap presenting Module-trait associations. Each row depicts a module eigengene, and each column represents a specific characteristic trait. (**E**): The weighted network of significant genes associated with the module identified by MEturquoise. The color and extent of the circle indicated the weight of each gene in the network, with circle size indicating the degree of corresponding genes in the network, indicative of gene importance. (**F**): Scatter plot of the most enriched KEGG pathways derived from genes associated with the regulatory network of *FL2–FL4* in *F. sinkiangensis*.

According to KOG annotation, we further identified three genes (*FL2–FL4*) associated with flowering in the genome of *F. sinkiangensis* (Figure 6A). We determined the expression levels of these three genes in the leaves of flowering and unflowering *F. sinkiangensis* using RT-qPCR (Figure 6A). As presented in Figure 6A, these genes in FLL were upregulated to reach higher expression levels, relative to UFLL, supporting their role in the flowering process of *F. sinkiangensis*. Given this function in the flowering of *F. sinkiangensis*, we developed a co-expression system utilizing WGCNA according to all transcriptomic assemblies for *F. sinkiangensis*. The expression data of all *F. sinkiangensis* genes were used for WGCNA development. We established a soft threshold of 732 (*R*^2^ = 0.85) to produce a scale-free network (Figure 6B). All biological replicates from the same group were separated from others, supporting the necessity of these three genes in flowering traits of *F. sinkiangensis* (Figure 6C). Three modules were found via hierarchical clustering and dynamic branch cleavage, and each module was assigned a unique identifying color (Figure 6D). The modules highly associated with related characteristics were removed for further regulatory pathway development of *FL2–FL4* in *F. sinkiangensis* leaves (Figure 6D). According to the WGCNA results, the MEturquoise module was associated with the expression level of *FL2*, *FL3,* and *FL4* (Figure 6D). Throughout genes derived from these modules, we produced co-expression regulatory systems for *FL2*, *FL3,* and *FL4* in *F. sinkiangensis* leaves (Figure 6E). We characterized six transcription factors that were responsible for the positive regulation of *FL2–FL4* expression in *F. sinkiangensis* (*p* < 0.05; Figure 6E). As presented in Figure 6E, *TGA*, *MYBP*, *WRKY33*, *MEF2A*, *HSFF*, and *K09264* were hub transcription factors that were responsible for regulating *FL2–FL4* expression in *F. sinkiangensis* and were positively correlated with the expression of *FL2–FL4* (Figure 6E). *WRKY*- and *MYB*-type transcription factors regulate the flowering process of numerous plants. Further KEGG pathway analysis demonstrated that the genes in the regulation network of *FL2–FL4* are mainly involved in Carbon metabolism, Porphyrin and chlorophyll metabolism, Phenylpropanoid biosynthesis, Sulfur relay system, Photosynthesis, Alanine, aspartate and glutamate metabolism, Riboflavin metabolism, Terpenoid backbone biosynthesis, Linolic acid biosynthesis, Hormone signaling pathway, Peroxisome, Glutathione metabolism, Flavonoid biosynthesis, Folate biosynthesis, and Phosphatidylinositol signaling system (Figure 6F). Phenylpropanoid biosynthesis, Photosynthesis, and Linolic acid biosynthesis were also significantly identified in the regulation network of *FL2–FL4*, demonstrating their importance in contributing to the flowering of *F. sinkiangensis* (Figure 6F). Overall, these results suggested that three flowering genes were responsible for regulating the flowering process of *F. sinkiangensis*, particularly six transcription factors with roles in Phenylpropanoid biosynthesis and Photosynthesis biosynthesis.

## 4. Discussion

Systemic investigation of the flowering mechanisms of *F. sinkiangensis* flowering is of great significance for cultivating high plants. In this study, we deployed transcriptome, proteome, and metabolome analysis to compare the multilayer differences in leaves and roots of *F. sinkiangensis* with flowering and unflowering phenotypes. We found that all variations in transcriptome, proteome, and metabolome in leaves were closely associated with the flowering traits of *F. sinkiangensis*. The Photosynthesis and Phenylpropanoid biosynthesis in *F. sinkiangensis* with flowering phenotype were more active, suggesting their involvement in mediating the flowering process of *F. sinkiangensis*. Additionally, we identified three genes encoding flowering genes in *F. sinkiangensis*, including *FL2–FL4*. The expression levels of *FL2*, *FL3,* and *FL4* in leaves of flowering plants were higher than those in unflowering plants. We identified six transcription factors responsible for regulating *FL2–FL4* expression in leaves of flowering *F. sinkiangensis* by developing the regulation network of *FL2–FL4*. Moreover, we identified that genes related to Photosynthesis and Phenylpropanoid biosynthesis also performed essential functions in the regulation network of *FL2–FL4* in leaves of flowering *F. sinkiangensis*, supporting their importance in the flowering process of *F. sinkiangensis*. Overall, our results elucidated the integrated flowering mechanism of *F. sinkiangensis* and provided a theoretical basis for cultivating *F. sinkiangensis* with high economic quality.

Plant flowering is an essential stage in plant growth and development that is related to the successful reproduction of plants and crop yield. *F.* spp. is a perennial flowering and fruiting plant that grows for 7–8 years before flowering [3]. We found that the flowering of *F.* spp. was closely associated with transcription and proteome changes in leaves, especially variations relevant to Photosynthesis and Phenylpropanoid biosynthesis. In our analysis, Phenylpropanoid metabolism was mainly involved in the flowering of *F.* spp., so it was speculated that Phenylpropanoids were also involved in the flowering pathway of *F.* spp. Typically, the Phenylpropanoid pathway was closely related to plant flowering [29]. *AtLOV1* overexpression changed the lignin content and cell wall monomer composition and delayed the flowering time [30]. Mutations of three key enzymes in the *A. thaliana* Phenylpropanoid biosynthesis pathway disrupted auxin transport in plants, delaying their flowering time [15].

Photosynthesis converts light energy into chemical energy and organic matter, enabling plants to grow and flower. Although sucrose induces flowering in different plants [31,32], the molecular mechanism remains unclear. The *miR172* is associated with the flowering induction of potato plants in a sucrose-dependent manner, and it is a downstream signaling component of *StSUT4* in regulating flowering [33]. Sucrose, together with ethylene, was confirmed to regulate the protein stability of GIGANTEA, which gates GA signaling by stabilizing DELLA [34,35]. In this study, Photosynthesis of *F.* spp. leaves and root systems were closely related to flowering traits. Many studies have shown that sucrose and SUT may regulate plant flowering through the GA pathway [12]. However, such a regulatory mechanism in the leaves and roots of *F.* spp. needs further verification.

In the plant life cycle, flower formation is regulated by both exogenous environmental signals and endogenous developmental signals [36]. Numerous transcription factors regulate these biological processes in plants, especially flowering [37]. The role of *WRKY* in regulating plant flowering has also been reported in several plant species. *AtWRKY12*, *AtWRKY13*, *AtWRKY71*, *AtWRKY75,* and other genes have been reported to be involved in the flowering regulation of *A. thaliana* [38]. Overexpression of *CpWRKY75* in *A. thaliana* significantly promoted the development time of the plants [39]. Overexpression of the OsMYB1R1-VP64 fusion protein in rice can significantly enhance yield and inhibit the expression of flowering-related genes, delaying flowering [40]. In this study, six transcription factors are involved in regulating the expression of flowering-related genes *FL2–FL4* in *F. sinkiangensis*. Numerous studies have found that *MYB* and *WRKY* can regulate Phenylpropane biosynthetic pathways in plants [41,42]. This offers a theoretical basis for the propagation of the *F.* spp. plant.

To further explore the mechanism of flowering regulation in *F. sinkiangensis,* a high-quality reference genome of *F. sinkiangensis* was needed in future research. Meanwhile, since the genetic transformation system in *F. sinkiangensis* has not yet been established, it is necessary to verify the function of key genes in flowering regulation by transforming *A. thaliana* or other plants [43]. In the future, the genetic transformation system of *F. sinkiangensis* may be established by referring to the cut-dip-budding (CBD) method [44] so as to make it possible to study functional genes in *F. sinkiangensis* and use the CBD method to solve the natural resources problem in *F. sinkiangensis*.

Overall, our study deciphered the transcription, proteomic, and metabolic landscape associated with the flowering of *F. sinkiangensis*, contributing to the cultivation of *F. sinkiangensis* seedlings. Additional research on the key genes of flowering traits will assist us in better understanding the molecular mechanism of flowering for *F.* spp.

## 5. Conclusions

Presently, our multi-omics analyses results showed that active Photosynthesis and Phenylpropanoid biosynthesis are involved in regulating the flowering phenotype in *F. sinkiangensis*. Three flowering-related genes *FL2–FL4* significantly correlate with six transcription factors, Photosynthesis and Phenylpropanoid biosynthesis in regulatory networks. This work provides a theoretical basis for further study on the mechanism of flowering regulation in *F. sinkiangensis.*

## Data Availability

The transcript expression abundances are available in the National Genomics Data Center, China National Center for Bioinformation/Beijing Institute of Genomics, Chinese Academy of Sciences that are publicly accessible at https://ngdc.cncb.ac.cn (PRJCA029527), accessed on 30 August 2024.

## Supplementary Materials

The following supporting information can be downloaded at: https://www.mdpi.com/article/10.3390/genes15101275/s1, Table S1: GO enrichment results of DEGs from FLR vs. UFLR; Table S2: KEGG pathway enrichment results of DEGs from FLR vs. UFLR; Table S3: GO enrichment results of DEGs from FLL vs. UFLL; Table S4: GO enrichment results of DEPs from FLR vs. UFLR; Table S5: KEGG pathway enrichment results of DEPs from FLR vs. UFLR.
